# MenAfriVac as an Antitetanus Vaccine

**DOI:** 10.1093/cid/civ512

**Published:** 2015-11-09

**Authors:** Ray Borrow, Yuxiao Tang, Ahmadu Yakubu, Prasad S. Kulkarni, F. Marc LaForce

**Affiliations:** 1 Vaccine Evaluation Unit, Public Health England, Manchester Royal Infirmary, United Kingdom; 2 Meningitis Vaccine Project, PATH, Seattle, Washington; 3 Immunization, Vaccines and Biologicals/Expanded Programme on Immunization, World Health Organization, Geneva, Switzerland; 4 Serum Institute of IndiaLtd, Pune

**Keywords:** group A meningococcal, PsA-TT, tetanus, conjugate vaccine

## Abstract

***Background.*** The group A meningococcal conjugate vaccine, PsA-TT, uses tetanus toxoid (TT) as a carrier protein (PsA-TT). TT as a carrier protein in other conjugate vaccines is known to be immunogenic and generates a robust anti-TT response.

***Methods.*** Clinical studies in Africa assessed whether PsA-TT generated tetanus serologic responses when tested in African populations (toddlers to adults). Second, the high acceptance of PsA-TT mass immunization campaigns in the 1- to 29-year age group meant that a sizeable fraction of women of reproductive age received PsA-TT. Incidence data for neonatal tetanus were reviewed for countries with and without PsA-TT campaigns to check whether this had any impact on the incidence.

***Results.*** PsA-TT generated robust tetanus serologic responses in 1- to 29-year-olds, similar to those expected after a booster dose of TT. Neonatal cases of tetanus fell by 25% in countries that completed PsA-TT campaigns in 1- to 29-year-olds.

***Conclusions.*** Although these data are not yet definitive, they are consistent with the hypothesis that improved community immunity to tetanus as a result of the PsA-TT campaigns may be having an impact on the incidence of neonatal tetanus in sub-Saharan Africa.

***Clinical Trials Registration.*** ISRCTN17662153 (PsA-TT 001); ISRTCN78147026 (PsA-TT 002); ISRCTN87739946 (PsA-TT 003); ISRCTN46335400 (PsA-TT 003a); ISRCTN82484612 (PsA-TT 004); CTRI/2009/091/000368 (PsA-TT 005); PACTR ATMR2010030001913177 (PsA-TT 006); and PACTR201110000328305 (PsA-TT 007).

Maternal and neonatal tetanus is still a significant, although vaccine-preventable, cause of morbidity and mortality in many developing countries. When tetanus develops, case-fatality rates are high and treatment is often limited by paucity of resources. The Maternal and Neonatal Tetanus Elimination Initiative, relaunched by the World Health Organization (WHO), United Nations Children's Fund, and United Nations Population Fund in 1999 following the initial launch in 1989, has made substantial progress in eliminating maternal and neonatal tetanus [1]. Although the number of deaths due to neonatal tetanus has fallen by 94%, from the estimated 787 000 in 1988 to 49 000 in 2013, further progress will require improved vaccination programs. WHO data indicate that sub-Saharan Africa remains one the highest-risk areas for neonatal and nonneonatal tetanus [1].

Maternal immunization with tetanus toxoid (TT)–containing vaccines has led to 82% of today's newborns being protected from tetanus [2]. For maternal protection, the degree and duration of immunity increase with the number of appropriately spaced TT doses administered. One dose of TT ensures little, if any, protection. Following a second dose, the mean antibody level usually exceeds the protective level of 0.01 IU/mL, although up to 10% may remain unprotected. Immunity declines over time, and after 1 year the percentage of those unprotected persons can be up to 20%. For this reason, a third dose of tetanus toxoid should be given during the subsequent pregnancy or 6–12 months after the initial 2 doses, giving a level of immunity that is high and which persists for at least 5 years. Even after the third dose, when given at yearly intervals, a fourth dose will give immunity for 10 years and a fifth dose immunity for at least 20 years [3].

In children, 3 primary doses of diphtheria-tetanus-pertussis (DTP) vaccine will induce a protective antibody level [4].

Glycoconjugate vaccines against *Haemophilus influenzae* type b (Hib) disease and various meningococcal and pneumococcal groups/types contain the specific bacterial poly/oligosaccharide conjugated to an immunogenic carrier protein, the most commonly utilized being either TT or the nontoxic mutant of diphtheria toxin (CRM197). These carrier proteins induce antibodies themselves, and for CRM_197_-induced antibodies, these have been shown to be functional [5].

During the development of PsA-TT, a choice of carrier protein had to be made. Discussions at the Meningitis Vaccine Project and its advisory bodies focused on published data on the efficacy of carrier proteins while asking whether there were public health advantages that could be attained with certain carrier molecules. Using TT as a carrier protein became an attractive option for 3 reasons: (1) Conjugate vaccines using TT as a carrier protein had been successfully developed; (2) both neonatal and nonneonatal tetanus were public health problems in sub-Saharan Africa; and (3) conjugate vaccines that were made with TT had shown an antitetanus serologic response when tested.

Thus, PsA-TT was developed by the Serum Institute of India, Ltd, and following extensive immunogenicity and safety trials was prequalified by WHO in 2010. Rollout of this vaccine is now well under way across the meningitis belt of sub-Saharan Africa and, as of the end of 2014, >210 million Africans between the ages of 1 and 29 years will have received a dose of PsA-TT [6]. This conjugate vaccine contains 10–33 µg of TT per dose or 3–9 Lf (limit of flocculation). This paper reviews the tetanus serologic data that were obtained during the phase 1–3 clinical trials as well as reported cases of tetanus in African countries before and after introduction of PsA-TT in large campaigns aimed at 1- to 29-year-olds.

## METHODS

### PsA-TT Trials

The trials that included TT serology are shown in Supplementary Table 1. PsA-TT-001 was a phase 1, double-blind, randomized study to evaluate the safety and immunogenicity of a single dose of PsA-TT vs a meningococcal polysaccharide A + C reference vaccine and a TT control vaccine, given as single intramuscular injections in healthy adults from 18 to 35 years of age [7]. PsA-TT-002 was a phase 2, observer-blind, randomized, active controlled study to compare the safety, immunogenicity, and induction of immunological memory of PsA-TT, a meningococcal ACWY polysaccharide vaccine and a Hib conjugate vaccine, administered in healthy toddlers 12–23 months of age [8]. PsA-TT-003 was a phase 2/3, observer-blind, randomized, active controlled study to compare the safety and immunogenicity of a single dose of PsA-TT with meningococcal ACWY polysaccharide vaccine administered in healthy subjects 2–29 years of age. PsA-TT-003a was a phase 2/3, observer-blind, randomized, active controlled study to compare the safety and immunogenicity of a single dose of PsA-TT with meningococcal ACWY polysaccharide vaccine administered in healthy subjects 2–10 years of age [9]. PsA-TT-005 was a phase 3, double-blind, randomized, active controlled study to evaluate the safety and consistency of immunogenicity of 3 consecutive lots of PsA-TT administered as a single dose to healthy children at 5–10 years of age. PsA-TT-007 was a phase 3, double-blind, randomized, controlled study to evaluate the immunogenicity and safety of different schedules and formulations of PsA-TT administered concomitantly with local Expanded Programme on Immunization (EPI) vaccines in healthy infants and toddlers. PsA-TT-001, -003a, and -005 were conducted in India; other studies were performed in Africa.

### Measurement of TT Immunoglobulin G

Baseline and postvaccination anti-TT immunoglobulin G (IgG) antibody concentrations were assessed in all the studies. PsA-TT-001, -002, -003, -003a, and -005 samples were assayed at the Immunoassay Laboratory at Public Health England's (formerly Health Protection Agency) Porton Down facility by standardized enzyme-linked immunosorbent assay (ELISA) [10, 11]. For each antigen, sera were titrated against known international standard sera, first International Tetanus reference serum 26/488. PsA-TT-007 samples were assayed at The Molecular Epidemiology Research Division of the University of Siena (EpidMol-UNISI) by a commercial TT IgG ELISA (DRG International Inc) [12]. Concentrations ≥0.1 IU/mL was considered protective, and the proportion of subjects with protective concentrations was calculated in terms of percentage. Geometric mean concentrations (GMCs) were calculated at each time point.

### Surveillance Data for Neonatal Tetanus

Surveillance data for neonatal tetanus cases in meningitis belt countries was obtained from the WHO website [13]. Tables were prepared listing neonatal tetanus cases and maternal TT2+ coverage from 2009 to 2013 for 3 categories of meningitis belt countries: (1) countries that had completed countrywide PsA-TT vaccination campaigns in 1- to 29-year-olds by 2012; (2) countries with either partial coverage with PsA-TT or with campaigns still proceeding; and (3) countries that had not yet begun PsA-TT campaigns (Table 1).

**Table 1.** Annual Reported Cases of Neonatal Tetanus and TT2 Coverage From 2009 to 2013 for Countries Completing Countrywide PsA-TT Campaigns in Persons Aged 1–29 Years by 2012

Country	Year of PsA-TT Campaign	2009		2010		2011		2012		2013	
		NT Cases	NT Coverage Estimate, %	NT Cases	NT Coverage Estimate, %	NT Cases	NT Coverage Estimate, %	NT Cases	NT Coverage Estimate, %	NT Cases	NT Coverage Estimate, %
Burkina Faso	2010	6	85	2	85	**2**	**88**	**1**	**88**	**0**	**88**
Mali	2010–2011	13	92	7	85	11	89	**10**	**89**	**12**	**85**
Niger	2010–2011	14	84	13	84	11	84	**3**	**84**	**1**	**81**
Gambia	2011	0	91	0	91	2	91	**0**	**92**	**0**	**82**
Chad	2012	146	60	279	60	215	60	225	43	**176**	**50**
Senegal	2012	16	88	12	88	21	88	14	91	**4**	**91**
Total		195		313		260		253		**193**	

Post–PsA-TT campaign data are shown in bold.

Abbreviations: NT, neonatal tetanus; PsA-TT, group A meningococcal conjugate vaccine; TT2, second dose of tetanus toxoid.

Using data from 2009 to 2013, the average annual numbers of cases of neonatal tetanus were tallied before and after countries had completed countrywide PsA-TT vaccination campaigns in 1- to 29-year-olds. Data from Nigeria were specifically excluded from the analysis because of a major increase in reported cases of neonatal tetanus in 2013, the reasons for which were unclear. For meningitis belt countries that had not yet begun PsA-TT campaigns, average annual neonatal tetanus cases for 2009–2011 were compared to 2011–2012 data. Similar comparisons were made for TT2 coverage in all countries.

## RESULTS

### PsA-TT-001

**Figure 1.**
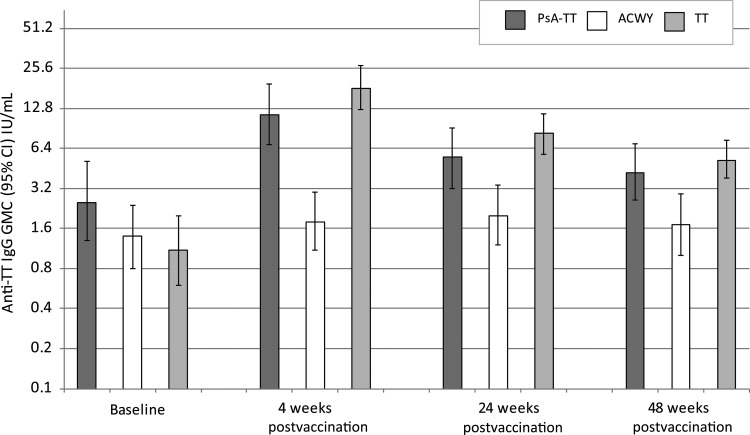
PsA-TT-001. Anti–tetanus toxoid (TT) immunoglobulin G (IgG) geometric mean concentrations (GMCs) (with 95% confidence interval [CI]) for healthy adults aged 18–35 years following vaccination with either meningococcal A conjugate vaccine, meningococcal ACWY polysaccharide vaccine, or TT vaccine.

At baseline, 95.7% (66/69) of healthy adults aged 18–35 years had protective anti-TT concentrations prior to vaccination. At 4 weeks following vaccination with either PsA-TT or TT, significantly higher anti-TT IgG GMCs were seen compared with those receiving AC polysaccharide vaccine (*P* < .0001 for both PsA-TT vs MenA + C and TT vs MenA + C) (Figure 1). These antibody levels persisted significantly higher at 24 (*P* = .0065 for PsA-TT vs MenA + C and *P* < .0001 for TT vs MenA + C) and 48 weeks (*P* = .0138 for PsA-TT vs MenA + C and *P* = .0006 for TT vs MenA + C). The anti-TT IgG GMCs did not significantly differ between the PsA-TT and TT groups over time.

### PsA-TT-002

**Figure 2.**
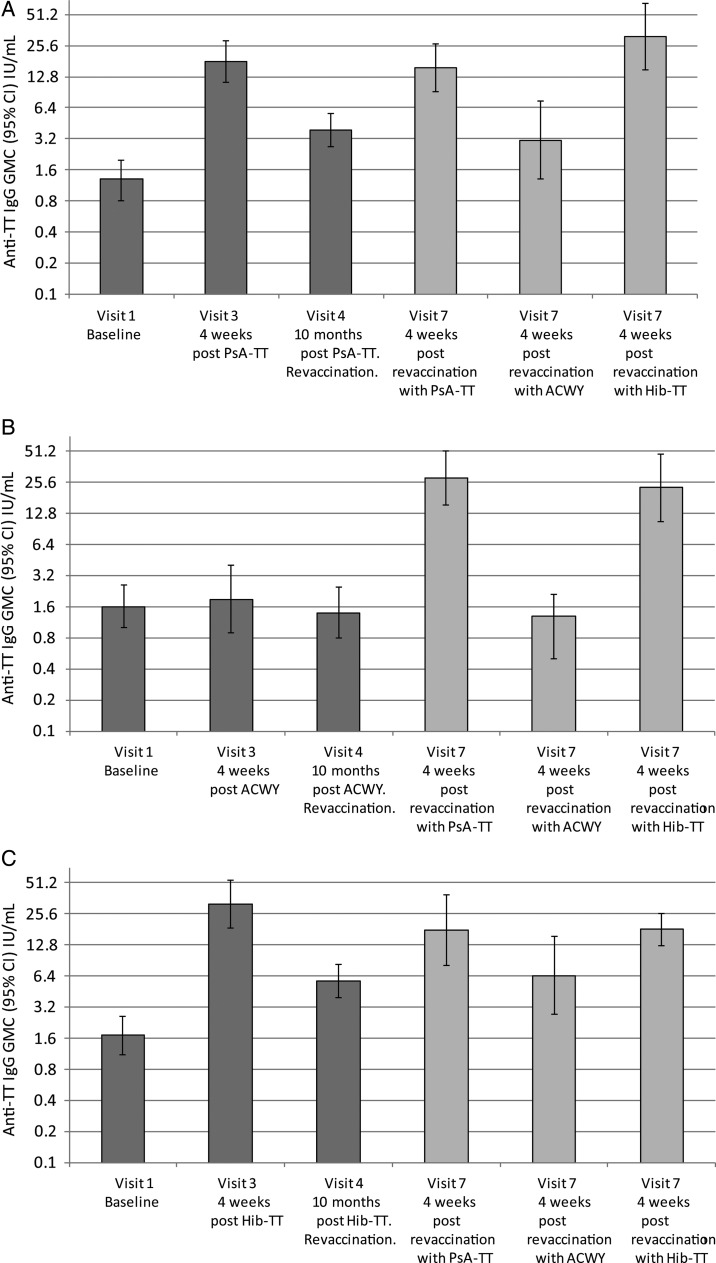
PsA-TT-002. Anti–tetanus toxoid (TT) immunoglobulin G (IgG) geometric mean concentrations (GMCs) (with 95% confidence interval [CI]) for healthy toddlers aged 12–23 months following vaccination with either meningococcal A conjugate vaccine (*A*), meningococcal ACWY polysaccharide vaccine (*B*), or *Haemophilus influenzae* (Hib) conjugate vaccine (*C*) and revaccination randomized 1:1:1 to receive either meningococcal A conjugate vaccine, a fifth of the dose of meningococcal ACWY polysaccharide vaccine, or a Hib conjugate vaccine.

A total of 99% (98/99) of healthy toddlers aged 12–23 months had protective anti-TT concentrations at baseline; by visit 3 and throughout the remainder of this study, this was at 100%. Figure 2 illustrates the anti-TT IgG GMCs by vaccine group. Primary vaccination with either PsA-TT or Hib-TT noticeably boosted anti-TT IgG GMCs, whereas no increase was seen following vaccination with ACWY vaccine. At 4 weeks after primary vaccination, anti-TT IgG GMC was significantly higher in the PsA-TT and Hib-TT groups than in ACWY group (*P* < .0001 for both PsA-TT vs ACWY and Hib-TT vs ACWY). Ten months following vaccination, anti-TT IgG GMCs had declined but remained significantly higher (*P* < .0001 for both PsA-TT vs ACWY and Hib-TT vs ACWY) in the 2 conjugate vaccine groups than the ACWY vaccine group. Revaccination with either PsA-TT or Hib-TT in those primed with PsA-TT or Hib-TT restored levels to that following the primary vaccination.

### PsA-TT-003 and PsA-TT-003a

**Figure 3.**
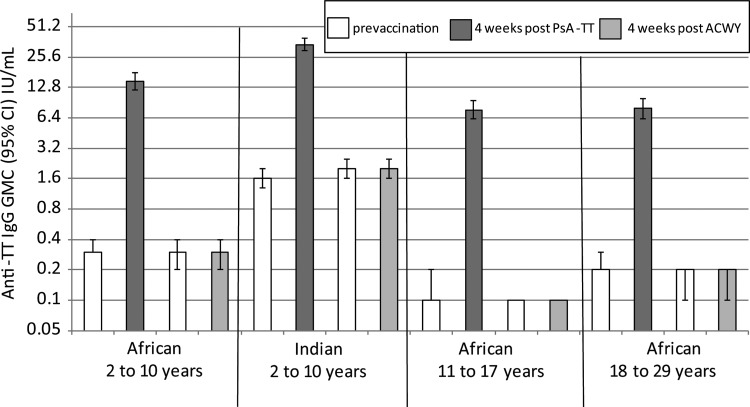
PsA-TT-003 and PsA-TT-003a. Anti–tetanus toxoid (TT) immunoglobulin G (IgG) geometric mean concentrations (GMCs) (with 95% confidence interval [CI]) for healthy Africans aged 2–10, 11–17, and 18–29 years and Indians aged 2–10 years following vaccination with either meningococcal A conjugate vaccine or meningococcal ACWY polysaccharide vaccine.

In healthy 2- to 10-year-old children, there was a significant difference in the percentage of children with anti-TT IgG concentrations ≥0.1 IU/mL at baseline between the African (76.6% [229/299]) and Indian (96.2% [327/340]) children (*P* < .0001). This was also reflected in the anti-TT GMCs (Figure 3). Four weeks following a dose of PsA-TT, anti-TT IgG GMCs were significantly higher in the Indian children as opposed to African children with GMCs of 34.1 IU/mL (95% confidence interval [CI], 29.7–39.1) and 14.7 IU/mL (95% CI, 12.0–18.0), respectively (*P* < .0001). Four weeks following a dose of PsA-TT, anti-TT IgG levels were significantly higher, regardless of age (*P* < .0001 for both Indian and African participants), compared with ACWY vaccine. Of note, at baseline, for those aged 11–17 years and 18–29 years, only 48.3% (143/296) and 63.6% (187/294), respectively, had anti-TT IgG concentrations ≥0.1 IU/mL.

### PsA-TT-005

The 3 consecutively produced lots of PsA-TT vaccine, when administered as a single dose in children 5–10 years of age, gave consistent anti-TT IgG GMCs 4 weeks following vaccination of 24.1 (95% CI, 19.6–29.6), 20.3 (95% CI, 14.9–27.7), and 25.3 (95% CI, 19.8–32.3) IU/mL. All subjects who were assayed for anti-TT IgG had antibody concentrations ≥0.1 IU/mL after vaccination.

### PsA-TT-007

**Figure 4.**
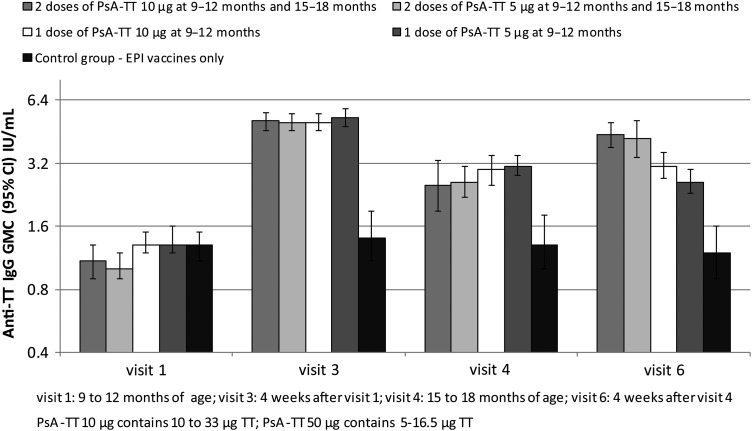
PsA-TT-007. Anti–tetanus toxoid (TT) immunoglobulin G (IgG) geometric mean concentrations (GMCs) (with 95% confidence interval [CI]) for healthy Malian infants immunized with either 1 or 2 doses of either a 10-µg or 5-µg dose of PsA-TT compared with a control group receiving only Expanded Programme on Immunization (EPI) vaccines.

In healthy 9- to 12-month-old Malian children, a single immunization with either a full or half dose of PsA-TT elicited a marked boost in anti-TT IgG GMCs. Four weeks following the immunization given to 9- to 12-month-olds, anti-TT IgG GMC was significantly higher in children who received PsA-TT than those who only received the EPI (measles and yellow fever) vaccines (*P* < .0001 for both a full dose of PsA-TT vs EPI only and a half-dose of PsA-TT vs EPI only). By 15 to 18 months of age, the anti-TT IgG GMCs remained significantly higher in children who received PsA-TT than those who had only received the EPI schedule (*P* = .0001 for a full dose of PsA-TT vs EPI only and *P* < .0001 for a half-dose of PsA-TT vs EPI only) (Figure 4). Four weeks following the immunization given at 15–18 months of age, anti-TT IgG GMC was increased in children who received a booster dose of PsA-TT, whereas the anti-TT IgG GMC did not change much in children who did not receive a booster dose of PsA-TT; anti-TT IgG GMC was significantly higher in children who received at least 1 vaccination of PsA-TT than those who only received the EPI vaccines (*P* < .0001 for at least 1 full dose of PsA-TT vs EPI only and for at least 1 half-dose of PsA-TT vs EPI only).

### Impact Data

Table 1 enumerates reported cases of neonatal tetanus for Burkina Faso, Mali, Niger, Gambia, Chad, and Senegal, countries that completed countrywide PsA-TT campaigns in 1- to 29-year-olds by 2012. Cases of neonatal tetanus in 2013 declined in 5 of the 6 countries, with Mali being the sole exception. When the average annual cases of neonatal tetanus in the 5 countries were compared before and after the PsA-TT campaigns (Table 2), there was a 25% reduction in neonatal tetanus cases (260 to 194). Maternal TT2 coverage was unchanged before and after the introduction campaigns (82.2% to 81.0%).

**Table 2.** Average Annual Cases of Neonatal Tetanus and TT2 Coverage in Meningitis Belt Countries That Have Introduced or Have Yet to Introduce PsA-TT

Campaign Area	Average Annual Cases of NT, No.			TT2 Coverage, %		
	2009–2011	2012–2013	% Change	2009–2011	2012–2013	% Change
No PsA-TT campaigns^a^	121.9	139.5	+14.4%	85.6	79.2	−7.4%
	Before campaign	After campaign	% change	Before campaign	After campaign	% change
Campaigns in part of country^b^	64	68	+6.5%	86.6	85.8	−0.9%
Campaigns covering all country^c^	260.2	194.5	−25.4%	82.2	81.0	−2.5%

Abbreviations: NT, neonatal tetanus; PsA-TT, group A meningococcal conjugate vaccine; TT2, second dose of tetanus toxoid.

^a^ Côte d'Ivoire, Ethiopia, Mauritania, Democratic Republic of Congo, Guinea, Central African Republic.

^b^ Cameroon, Togo, Benin, Ghana.

^c^ Burkina Faso, Mali, Niger, Gambia, Chad, Senegal.

Average annual cases of neonatal tetanus in countries that had mounted regional PsA-TT campaigns (Table 3) were unchanged (64 to 68 cases) before and after the campaigns. Not unexpectedly, protection at birth coverage estimate remained the same before and after the regional campaigns (86.6% to 85.8%).

**Table 3.** Annual Reported Cases of Neonatal Tetanus and TT2 Coverage for Countries Completing Regional PsA-TT Campaigns in 1- to 29-Year-Olds by 2012

Country	Partial Campaign Year	2009		2010		2011		2012		2013	
		NT Cases	NT Coverage Estimate, %	NT Cases	NT Coverage Estimate, %	NT Cases	NT coverage Estimate, %	NT Cases	NT Coverage Estimate, %	NT Cases	NT Coverage Estimate, %
Cameroon	2011	39	91	29	91	31	**75**	**23**	**85**	**43**	**85**
Togo	2012	17	81	28	81	28	81	20	81	**26**	**77**
Benin	2012	4	92	3	92	6	92	4	93	**8**	**93**
Ghana	2012	8	86	1	86	5	88	0	88	**1**	**88**
Total		68		61		70		47		78	

Post–PsA-TT campaign data are shown in bold.

Abbreviations: NT, neonatal tetanus; PsA-TT, group A meningococcal conjugate vaccine; TT2, second dose of tetanus toxoid.

For the 5 countries (Côte d'Ivoire, Ethiopia, Mauritania, Democratic Republic of Congo, and Guinea) that had not yet mounted PsA-TT campaigns (Table 4), the average number of reported cases of neonatal tetanus had increased from 121.9 (2009–2011) to 139.5 (2012–2013), and protection at birth coverage had fallen from 85.6% (2009–2011) to 79.2% (2012–2013).

**Table 4.** Annual Reported Cases of Neonatal Tetanus and TT2 Coverage From 2009 to 2013 for Meningitis Belt Countries That Have Not Done Regional or Countrywide PsA-TT Campaigns in Persons Aged 1–29 Years by 2012

Country	2009		2010		2011		2012		2013	
	NT Cases	NT Coverage Estimate, %	NT Cases	NT Coverage Estimate, %	NT Cases	NT Coverage Estimate, %	NT Cases	NT Coverage Estimate, %	NT Cases	NT Coverage Estimate, %
Côte d'Ivoire	8	92	3	82	3	82	9	82	15	82
Ethiopia	50	88	75	88	33	88	40	68	16	72
Mauritania	2	87	1	87	1	80	0	80	0	80
Republic of Congo	2	82	2	83	0	83	2	83	5	83
Guinea	21	96	64	90	17	80	8	80	51	80
Central African Republic	15	86	20	86	49	80	65	80	68	80
Total	98		165		103		124		155	

Abbreviations: NT, neonatal tetanus; PsA-TT, group A meningococcal conjugate vaccine; TT2, second dose of tetanus toxoid.

## DISCUSSION

In all age groups and in all countries studied, PsA-TT significantly boosted anti-TT IgG levels. The magnitude of the response was not significantly different compared with a full dose of TT vaccine in healthy Indian adults aged 18–35 years and a Hib-TT vaccine in healthy African toddlers aged 12–23 months. Given the low percentage (48.3%) of 11- to 17-year-old Africans, recruited from Mali, Senegal, and the Gambia, with anti-TT IgG concentrations ≥0.1 IU/mL, an extra booster dose of TT is of huge benefit. as many of these subjects will go on to be future mothers. One striking difference in baseline anti-TT IgG concentrations was demonstrated in the 2- to 10-year age group where Indian children had significantly higher levels than African children. One explanation for this difference lies in the fact that the Indian Universal Immunization Programme recommends at least 7 doses of TT: 3 doses of DTP in infancy, 2 booster doses at 16–24 months, 5–6 years of age, and at 10 and 16 years of age, as opposed to the 3-dose EPI schedule in Africa [14, 15].

The neonatal tetanus surveillance data from meningitis belt countries are interesting but incomplete. In countries that have mounted national campaigns, there has been a decrease in reported cases of 25%. This has not been noted in countries that have not yet mounted campaigns or in countries that had partial campaigns. Neonatal tetanus is an underreported disease, and caution needs to be taken in overinterpreting surveillance data. Nonetheless, the information from countries that have completed PsA-TT campaigns is encouraging and is consistent with the tetanus serologic data from the PsA-TT clinical trials in Africa. Increasing immunity to tetanus in women of reproductive age doubtlessly happened at a result of the introduction of PsA-TT in 1- to 29-year-olds, but the extent to which this has translated to decreases in the incidence of neonatal tetanus needs to be further quantified.

## Supplementary Data

Supplementary materials are available at *Clinical Infectious Diseases* online (http://cid.oxfordjournals.org). Supplementary materials consist of data provided by the author that are published to benefit the reader. The posted materials are not copyedited. The contents of all supplementary data are the sole responsibility of the authors. Questions or messages regarding errors should be addressed to the author.

Supplementary Data
