# Supplementary material for: MenAfriVac as an Antitetanus Vaccine
Source: Clin Infect Dis. 2015 Nov 9;61(Suppl 5):S570–7. doi: 10.1093/cid/civ512 (PMC4639489; doi:10.1093/cid/civ512)
Supplement: Supplementary Data [file supp_61_suppl-5_S570__index.html]

Supplementary Data 

# MenAfriVac as an Antitetanus Vaccine

## Supplementary Data

Supplementary Data

- Supplementary Table 1 - docx file
- Supplementary Table 2 - docx file
- Supplementary Table 3 - docx file
- Supplementary Table 4 - docx file
- Supplementary Table 5 - docx file
- Supplementary Table 6 - docx file
