# Supplementary material for: MenAfriVac as an Antitetanus Vaccine
Source: Clin Infect Dis. 2015 Nov 9;61(Suppl 5):S570–7. doi: 10.1093/cid/civ512 (PMC4639489; doi:10.1093/cid/civ512)
Supplement: Supplementary Data [file supp_civ512_civ512supp_table1.docx]

| **Supplementary Table 1** | | | | |
| --- | --- | --- | --- | --- |
| **PsA-TT-001. A Phase I, double-blind, randomized study to evaluate the safety and immunogenicity of a new meningococcal A conjugate vaccine versus a meningococcal polysaccharide A+C reference vaccine and a tetanus toxoid control vaccine, given as single intramuscular injections in healthy adults from 18 to 35 years of age.** | | | | |
| Summary of percentage of subjects with Tetanus toxoid IgG concentration ≥ 0.1 IU/mL – ITT population | | | | |
| Visit | Statistic | PsA-TT | Men A+C | Tetanus Toxoid |
| Visit 1^a^ | N (Missing) | 22 (2) | 24 (1) | 23 (2) |
|  | n (%) | 21 (95.5) | 23 (95.8) | 22 (95.7) |
|  | 95% CI | (77.2, 99.9) | (78.9, 99.9) | (78.1, 99.9) |
| Visit 4^b^ | N (Missing) | 24 (0) | 25 (0) | 25 (0) |
|  | n (%) | 23 (95.8) | 25 (100.0) | 25 (100.0) |
|  | 95% CI | (78.9, 99.9) | (86.3, 100.0) | (86.3, 100.0) |
| Visit 5^c^ | N (Missing) | 23 (1) | 23 (2) | 25 (0) |
|  | n (%) | 22 (95.7) | 23 (100.0) | 25 (100.0) |
|  | 95% CI | (78.1, 99.9) | (85.2, 100.0) | (86.3, 100.0) |
| Visit 6^d^ | N (Missing) | 24 (0) | 25 (0) | 25 (0) |
|  | n (%) | 23 (95.8) | 25 (100.0) | 25 (100.0) |
|  | 95% CI | (78.9, 99.9) | (86.3, 100.0) | (86.3, 100.0) |
| Summary of geometric mean concentration (GMC) of Tetanus toxoid IgG concentration – ITT population | | | | |
| Visit 1 | N (Missing) | 22 (2) | 24 (1) | 23 (2) |
|  | GMC | 2.5 | 1.4 | 1.1 |
|  | 95% CI | (1.3, 5.1) | (0.8, 2.4) | (0.6, 2.0) |
| Visit 4 | N (Missing) | 24 (0) | 25 (0) | 25 (0) |
|  | GMC | 11.5 | 1.8 | 18.2 |
|  | 95% CI | (6.8, 19.5) | (1.1, 3.0) | (12.6, 26.9) |
| Visit 5 | N (Missing) | 23 (1) | 23 (2) | 25 (0) |
|  | GMC | 5.5 | 2.0 | 8.3 |
|  | 95% CI | (3.2, 9.1) | (1.2, 3.4) | (5.8, 11.7) |
| Visit 6 | N (Missing) | 24 (0) | 25 (0) | 25 (0) |
|  | GMC | 4.2 | 1.7 | 5.2 |
|  | 95% CI | (2.6, 6.9) | (1.0, 2.9) | (3.8, 7.4) |

^a^ screening, 1 week prior to vaccination

^b^4 weeks after vaccination

^c^24 weeks after vaccination

^d^48 weeks after vaccination
