# Supplementary material for: MenAfriVac as an Antitetanus Vaccine
Source: Clin Infect Dis. 2015 Nov 9;61(Suppl 5):S570–7. doi: 10.1093/cid/civ512 (PMC4639489; doi:10.1093/cid/civ512)
Supplement: Supplementary Data [file supp_civ512_civ512supp_table2.docx]

| **Supplementary Table 2** | | | | | | | | | | | | | |
| --- | --- | --- | --- | --- | --- | --- | --- | --- | --- | --- | --- | --- | --- |
| **PsA-TT-002. A Phase II, observer-blind, randomized, active controlled study to compare the safety, immunogenicity, and induction of immunological memory of a meningococcal A conjugate vaccine, a meningococcal ACWY polysaccharide vaccine and a Hib conjugate vaccine, administered in healthy toddlers 12–23 months of age.** | | | | | | | | | | | | | |
| Summary of Percentage of Subjects with Anti-TT IgG ELISA Concentrations ≥ 0.1 IU/mL at Visit 1, 3 and 4 for Primary Vaccine Groups, overall and by site - ITT Population | | | | | | | | | | | | | |
| Visit | | | Statistic | | PsA-TT | | PsACWY | | Hib-TT | | | | |
| Visit 1^a^ | | | N (Missing) | | 31 (0) | | 35 (0) | | 33 (0) | | | | |
|  |  |  | n (%) | | 31 (100.0) | | 34 (97.1) | | 33 (100.0) | | | | |
|  |  |  | 95% CI | | (88.8 , 100.0) | | (85.1 , 99.9) | | (89.4 , 100.0) | | | | |
| Visit 3^b^ | | | N (Missing) | | 18 (13) | | 21 (14) | | 20 (13) | | | | |
|  |  |  | n (%) | | 18 (100.0) | | 21 (100.0) | | 20 (100.0) | | | | |
|  |  |  | 95% CI | | (81.5 , 100.0) | | (83.9 , 100.0) | | (83.2 , 100.0) | | | | |
| Visit 4^c^ | | | N (Missing) | | 31 (0) | | 35 (0) | | 32 (1) | | | | |
|  |  |  | n (%) | | 31 (100.0) | | 34 (97.1) | | 32 (100.0) | | | | |
|  |  |  | 95% CI | | (88.8 , 100.0) | | (85.1 , 99.9) | | (89.1 , 100.0) | | | | |
| Summary of Percentage of Subjects with Anti-TT IgG ELISA Concentrations ≥ 0.1 IU/mL at Visit 4 and Visit 7 for Booster Vaccine Groups, overall and by site - ITT Population | | | | | | | | | | | | | |
| Visit | | Statistic | P-P (1a) | P-M (1b) | P-H (1c) | M-P (2a) | M-M (2b) | M-H (2c) | H-P (3a) | H-M (3b) | | H-H (3c) | |
| Visit 4 | | N (Missing) | 13 (0) | 11 (0) | 7 (0) | 10 (0) | 11 (0) | 14 (0) | 12 (0) | 8 (1) | | 12 (0) | |
|  |  | n (%) | 13 (100.0) | 11 (100.0) | 7 (100.0) | 10 (100.0) | 11 (100.0) | 13 (92.9) | 12 (100.0) | 8 (100.0) | | 12 (100.0) | |
|  |  | 95% CI | (75.3 , 100.0) | (71.5 , 100.0) | (59.0 , 100.0) | (69.2 , 100.0) | (71.5 , 100.0) | (66.1 , 99.8) | (73.5 , 100.0) | (63.1 , 100.0) | | (73.5 , 100.0) | |
| Visit 7^d^ | | N (Missing) | 12 (1) | 11 (0) | 7 (0) | 10 (0) | 11 (0) | 14 (0) | 11 (1) | 9 (0) | | 11 (1) | |
|  |  | n (%) | 12 (100.0) | 11 (100.0) | 7 (100.0) | 10 (100.0) | 11 (100.0) | 14 (100.0) | 11 (100.0) | 9 (100.0) | | 11 (100.0) | |
|  |  | 95% CI | (73.5 , 100.0) | (71.5 , 100.0) | (59.0 , 100.0) | (69.2 , 100.0) | (71.5 , 100.0) | (76.8 , 100.0) | (71.5 , 100.0) | (66.4 , 100.0) | | (71.5 , 100.0) | |
| Summary of Geometric Mean Concentrations (GMC) of Anti-TT IgG ELISA Concentrations at Visit 1 and Visit 3 for Primary Vaccine Groups, overall and by site - ITT Population | | | | | | | | | | | | | |
| Visit | | | | Statistic | | PsA-TT | | PsACWY | | Hib-TT | | | |
| Visit 1 | | | | N (Missing) | | 31 (0) | | 35 (0) | | 33 (0) | | | |
|  |  |  |  | GMC | | 1.3 | | 1.6 | | 1.7 | | | |
|  |  |  |  | 95% CI | | (0.8 , 2.0) | | (1.0 , 2.6) | | (1.1 , 2.6) | | | |
| Visit 3 | | | | N (Missing) | | 18 (13) | | 21 (14) | | 20 (13) | | | |
|  |  |  |  | GMC | | 18.0 | | 1.9 | | 31.5 | | | |
|  |  |  |  | 95% CI | | (11.3 , 28.7) | | (0.9 , 4.0) | | (18.6 , 53.2) | | | |
| Visit 4 | | | | N (Missing) | | 31 (0) | | 35 (0) | | 32 (1) | | | |
|  |  |  |  | GMC | | 3.9 | | 1.4 | | 5.7 | | | |
|  |  |  |  | 95% CI | | (2.7 , 5.6) | | (0.8 , 2.5) | | (3.9 , 8.2) | | | |
| Summary of Geometric Mean Concentrations (GMC) of Anti-TT IgG ELISA Concentrations at Visit 4 and Visit 7 for Booster Vaccine Groups, overall and by site - ITT Population | | | | | | | | | | | | | |
| **Visit** |  | **Statistic** | **P-P (1a)** | **P-M (1b)** | **P-H (1c)** | **M-P (2a)** | **M-M (2b)** | **M-H (2c)** | **H-P (3a)** | | **H-M (3b)** | | **H-H (3c)** |
| Visit 4 | | n | 13 (0) | 11 (0) | 7 (0) | 10 (0) | 11 (0) | 14 (0) | 12 (0) | | 8 (1) | | 12 (0) |
|  |  | GMC | 4.0 | 3.6 | 4.2 | 1.6 | 1.3 | 1.4 | 4.5 | | 5.6 | | 7.1 |
|  |  | 95% CI | (2.2 , 7.1) | (1.5 , 8.5) | (2.3 , 7.7) | (0.8 , 3.0) | (0.5 , 3.2) | (0.4 , 4.9) | (1.9 , 10.6) | | (2.4 , 13.0) | | (4.6 , 11.2) |
| Visit 7 | | n | 12 (1) | 11 (0) | 7 (0) | 10 (0) | 11 (0) | 14 (0) | 11 (1) | | 9 (0) | | 11 (1) |
|  |  | GMC | 15.7 | 3.1 | 31.2 | 27.9 | 1.3 | 22.6 | 17.6 | | 6.4 | | 18.0 |
|  |  | 95% CI | (9.2 , 26.9) | (1.3 , 7.4) | (14.8 , 65.6) | (15.4 , 50.8) | (0.5 , 3.2) | (10.6 , 48.1) | (8.0 , 38.8) | | (2.7 , 15.4) | | (12.6 , 25.5) |

^a^ Prior to primary vaccination

^b^4 weeks after primary vaccination

^c^Booster vaccination at 10 months after primary vaccination

^d^4 weeks after booster vaccination
