# Supplementary material for: MenAfriVac as an Antitetanus Vaccine
Source: Clin Infect Dis. 2015 Nov 9;61(Suppl 5):S570–7. doi: 10.1093/cid/civ512 (PMC4639489; doi:10.1093/cid/civ512)
Supplement: Supplementary Data [file supp_civ512_civ512supp_table3.docx]

| **Supplementary Table 3** | | | | | | | | | | |
| --- | --- | --- | --- | --- | --- | --- | --- | --- | --- | --- |
| **PsA-TT-003. A Phase II/III, observer-blind, randomized, active controlled study to compare the safety and immunogenicity of a meningococcal A conjugate vaccine (PsA-TT) with meningococcal ACWY polysaccharide vaccine administered in healthy subjects 2 to 29 years of age.** | | | | | | | | | | |
| Summary of Percentage of Subjects with Anti-TT IgG ELISA Concentrations ≥ 0.1 IU/ml at Visit 1 and Visit 3, overall and by site - ITT Population | | | | | | | | | | |
|  | | | **2-10(yrs.)** | | **11-17(yrs.)** | | **18-29(yrs.)** | | **Total** | |
| **Visit** | | **Statistic** | **PsA-TT** | **PsACWY** | **PsA-TT** | **PsACWY** | **PsA-TT** | **PsACWY** | **PsA-TT** | **PsACWY** |
| Visit 1^a^ | | N (Missing) | 202 (1) | 97 (2) | 198 (4) | 98 (1) | 198 (1) | 96 (2) | 598 (6) | 291 (5) |
|  |  | n (%) | 156 (77.2) | 73 (75.3) | 97 (49.0) | 46 (46.9) | 133 (67.2) | 54 (56.3) | 386 (64.5) | 173 (59.5) |
|  |  | 95% CI | (70.8, 82.8) | (65.5, 83.5) | (41.8, 56.2) | (36.8, 57.3) | (60.2, 73.7) | (45.7, 66.4) | (60.6, 68.4) | (53.6, 65.1) |
| Visit 3^b^ | | N (Missing) | 202 (1) | 97 (2) | 201 (1) | 98 (1) | 197 (2) | 94 (4) | 600 (4) | 289 (7) |
|  |  | n (%) | 201 (99.5) | 69 (71.1) | 200 (99.5) | 45 (45.9) | 189 (95.9) | 49 (52.1) | 590 (98.3) | 163 (56.4) |
|  |  | 95% CI | (97.3, 100.0) | (61.0, 79.9) | (97.3, 100.0) | (35.8, 56.3) | (92.2, 98.2) | (41.6, 62.5) | (97.0, 99.2) | (50.5, 62.2) |
| Summary of Geometric Mean Concentrations (GMC) of Anti-TT IgG ELISA Concentrations at Visit 1 and Visit 3, overall and by site - ITT Population | | | | | | | | | | |
|  |  | | **2-10(yrs.)** | | **11-17(yrs.)** | | **18-29(yrs.)** |  | **Total** | |
| **Visit** | **Statistic** | | **PsA-TT** | **PsACWY** | **PsA-TT** | **PsACWY** | **PsA-TT** | **PsACWY** | **PsA-TT** | **PsACWY** |
| Visit 1 | N (Missing) | | 202 (1) | 97 (2) | 198 (4) | 98 (1) | 198 (1) | 96 (2) | 598 (6) | 291 (5) |
|  | GMC | | 0.3 | 0.3 | 0.1 | 0.1 | 0.2 | 0.2 | 0.2 | 0.2 |
|  | 95% CI | | (0.3, 0.4) | (0.2, 0.4) | (0.1, 0.2) | (0.1, 0.1) | (0.2, 0.3) | (0.1, 0.2) | (0.2, 0.3) | (0.2, 0.2) |
| Visit 3 | N (Missing) | | 202 (1) | 97 (2) | 201 (1) | 98 (1) | 197 (2) | 94 (4) | 600 (4) | 289 (7) |
|  | GMC | | 14.7 | 0.3 | 7.7 | 0.1 | 7.9 | 0.2 | 9.7 | 0.2 |
|  | 95% CI | | (12.0, 18.0) | (0.2, 0.4) | (6.3, 9.4) | (0.1, 0.1) | (6.2, 10.0) | (0.1, 0.2) | (8.5, 10.9) | (0.2, 0.2) |

^a^Prior to vaccination

^b^4 weeks after vaccination
