# Supplementary material for: MenAfriVac as an Antitetanus Vaccine
Source: Clin Infect Dis. 2015 Nov 9;61(Suppl 5):S570–7. doi: 10.1093/cid/civ512 (PMC4639489; doi:10.1093/cid/civ512)
Supplement: Supplementary Data [file supp_civ512_civ512supp_table4.docx]

| **Supplementary Table 4** | | | | |
| --- | --- | --- | --- | --- |
| **PsA-TT-003a. A Phase II/III, observer-blind, randomized, active controlled study to compare the safety and immunogenicity of a meningococcal A conjugate vaccine (PsA-TT) with meningococcal ACWY polysaccharide vaccine administered in healthy subjects 2 to 10 years of age.** | | | | |
| Summary of Percentage of Subjects with Anti-TT IgG ELISA Concentrations ≥ 0.1 IU/mL at Visit 1 and Visit 3 - ITT Population | | | | |
| Visit | Statistic | | PsA-TT | PsACWY |
| Visit 1^a^ | N (Missing) | | 169 (0) | 171 (0) |
|  | n (%) | | 162 (95.9) | 165 (96.5) |
|  | 95% CI | | (91.7, 98.3) | (92.5, 98.7) |
| Visit 3^b^ | N (Missing) | | 168 (1) | 169 (2) |
|  | n (%) | | 168 (100.0) | 163 (96.4) |
|  | 95% CI | | (97.8, 100.0) | (92.4, 98.7) |
| Summary of Geometric Mean Concentrations (GMC) of Anti-TT IgG ELISA Concentrations at Visit 1 and Visit 3 - ITT Population | | | | |
| Visit | | Statistic | PsA-TT | PsACWY |
| Visit 1 | | N (Missing) | 169 (0) | 171 (0) |
|  |  | GMC | 1.6 | 2.0 |
|  |  | 95% CI | (1.3, 2.0) | (1.6, 2.5) |
| Visit 3 | | N (Missing) | 168 (1) | 169 (2) |
|  |  | GMC | 34.1 | 2.0 |
|  |  | 95% CI | (29.7, 39.1) | (1.6, 2.5) |

^a^Prior to vaccination

^b^4 weeks after vaccination
