# Supplementary material for: MenAfriVac as an Antitetanus Vaccine
Source: Clin Infect Dis. 2015 Nov 9;61(Suppl 5):S570–7. doi: 10.1093/cid/civ512 (PMC4639489; doi:10.1093/cid/civ512)
Supplement: Supplementary Data [file supp_civ512_civ512supp_table5.docx]

| **Supplementary Table 5** | | | | | |
| --- | --- | --- | --- | --- | --- |
| **PsA-TT-005. A Phase III, double-blind, randomized, active controlled study to evaluate the safety and consistency of immunogenicity of three consecutive lots of a meningococcal A conjugate vaccine administered as a single dose to healthy children at 5–10 years of age.** | | | | | |
| Summary of Percentage of Subjects with Anti-TT IgG ELISA Concentrations ≥ 0.1 IU/mL at Visit 1 and Visit 3 - ITT Population | | | | | |
| Visit | Statistic | Group 1A^a^ | Group 1B^b^ | Group 1C^c^ | PsAC |
| Visit 1^d^ | N (Missing) | 38 (0) | 37 (0) | 38 (0) | 19 (0) |
|  | n (%) | 36 (94.7) | 35 (94.6) | 37 (97.4) | 19 (100.0) |
|  | 95% CI | (82.3, 99.4) | (81.8, 99.3) | (86.2, 99.9) | (82.4, 100.0) |
| Visit 3^e^ | N (Missing) | 38 (0) | 37 (0) | 38 (0) | 19 (0) |
|  | n (%) | 38 (100.0) | 37 (100.0) | 38 (100.0) | 19 (100.0) |
|  | 95% CI | (90.7, 100.0) | (90.5, 100.0) | (90.7, 100.0) | (82.4, 100.0) |
| Summary of Geometric Mean Concentrations (GMC) of Anti-TT IgG ELISA Concentrations at Visit 1 and Visit 3 - ITT Population | | | | | |
| Visit | Statistic | Group 1A | Group 1B | Group 1C | PsAC |
| Visit 1 | N (Missing) | 38 (0) | 37 (0) | 38 (0) | 19 (0) |
|  | GMC | 2.5 | 2.5 | 2.5 | 2.8 |
|  | 95% CI | (1.4, 4.5) | (1.4, 4.5) | (1.6, 3.9) | (1.7, 4.7) |
| Visit 3 | N (Missing) | 38 (0) | 37 (0) | 38 (0) | 19 (0) |
|  | GMC | 24.1 | 20.3 | 25.3 | 2.3 |
|  | 95% CI | (19.6, 29.6) | (14.9, 27.7) | (19.8, 32.3) | (1.4, 4.0) |

^a^subjects received PsA-TT vaccine from Lot A

^b^subjects received PsA-TT vaccine from Lot B

^c^subjects received PsA-TT vaccine from Lot C

^d^Prior to vaccination

^e^4 weeks after vaccination
