# Supplementary material for: MenAfriVac as an Antitetanus Vaccine
Source: Clin Infect Dis. 2015 Nov 9;61(Suppl 5):S570–7. doi: 10.1093/cid/civ512 (PMC4639489; doi:10.1093/cid/civ512)
Supplement: Supplementary Data [file supp_civ512_civ512supp_table6.docx]

| **Supplementary Table 6** | | | | | | |
| --- | --- | --- | --- | --- | --- | --- |
| **PsA-TT-007. A Phase 3, double-blind, randomized, controlled study to evaluate the immunogenicity and safety of different schedules and formulations of a meningococcal A conjugate vaccine administered concomitantly with local EPI vaccines in healthy infants and toddlers** | | | | | | |
| Summary of percentage of subjects with Tetanus toxoid-specific IgG concentration ≥ 0.1 IU/ml – ITT population | | | | | | |
| Visit | Statistics | Group 1A^a^ | Group 1B^b^ | Group 2A^c^ | Group 2B^d^ | Group 3^e^ |
| Visit 1^f^ | N (Missing) | 48 (2) | 49 (1) | 47 (3) | 47 (3) | 49 (1) |
|  | n (%) | 48 (100.0) | 49 (100.0) | 47 (100.0) | 47 (100.0) | 49 (100.0) |
|  | 95% CI | (93.9, 100.0) | (94.1, 100.0) | (93.8, 100.0) | (93.8, 100.0) | (94.1, 100.0) |
| Visit 3^g^ | N (Missing) | 48 (2) | 50 (0) | 47 (3) | 49 (1) | 50 (0) |
|  | n (%) | 48 (100.0) | 50 (100.0) | 47 (100.0) | 49 (100.0) | 49 (98.0) |
|  | 95% CI | (93.9, 100.0) | (94.2, 100.0) | (93.8, 100.0) | (94.1, 100.0) | (89.4, 99.9) |
| Visit 4^h^ | N (Missing) | 48 (2) | 50 (0) | 49 (1) | 49 (1) | 46 (4) |
|  | n (%) | 47 (97.9) | 50 (100.0) | 49 (100.0) | 49 (100.0) | 45 (97.8) |
|  | 95% CI | (88.9, 99.9) | (94.2, 100.0) | (94.1, 100.0) | (94.1, 100.0) | (88.5, 99.9) |
| Visit 6^i^ | N (Missing) | 47 (3) | 50 (0) | 49 (1) | 49 (1) | 45 (5) |
|  | n (%) | 47 (100.0) | 49 (98.0) | 49 (100.0) | 49 (100.0) | 45 (100.0) |
|  | 95% CI | (93.8, 100.0) | (89.4, 99.9) | (94.1, 100.0) | (94.1, 100.0) | (93.6, 100.0) |
| Summary of geometric mean concentration (GMC) of Tetanus toxoid-specific IgG concentration – ITT population | | | | | | |
| Visit | Statistics | Group 1A | Group 1B | Group 2A | Group 2B | Group 3 |
| Visit 1 | N (Missing) | 48 (2) | 49 (1) | 47 (3) | 47 (3) | 49 (1) |
|  | GMC | 1.1 | 1.0 | 1.3 | 1.3 | 1.3 |
|  | 95% CI | (0.9, 1.3) | (0.9, 1.2) | (1.2, 1.5) | (1.2, 1.6) | (1.1, 1.5) |
| Visit 3 | N (Missing) | 48 (2) | 50 (0) | 47 (3) | 49 (1) | 50 (0) |
|  | GMC | 5.1 | 5.0 | 5.2 | 5.3 | 1.4 |
|  | 95% CI | (4.6, 5.6) | (4.6, 5.5) | (4.7, 5.8) | (4.8, 5.8) | (1.1, 1.9) |
| Visit 4 | N (Missing) | 48 (2) | 50 (0) | 49 (1) | 49 (1) | 46 (4) |
|  | GMC | 2.5 | 2.6 | 3.0 | 3.1 | 1.3 |
|  | 95% CI | (1.9, 3.3) | (2.2, 2.9) | (2.5, 3.5) | (2.8, 3.5) | (1.0, 1.8) |
| Visit 6 | N (Missing) | 47 (3) | 50 (0) | 49 (1) | 49 (1) | 45 (5) |
|  | GMC | 4.4 | 4.2 | 3.1 | 2.6 | 1.2 |
|  | 95% CI | (3.8, 5.0) | (3.4, 5.1) | (2.7, 3.6) | (2.3, 3.0) | (0.9, 1.6) |

^a^subjects received two doses of PsA-TT 10 µg at 9 to 12 months and 15 to 18 months of age and EPI vaccines;

^b^subjects received two doses of PsA-TT 5 µg at 9 to 12 months and 15 to 18 months of age and EPI vaccines^;^

^c^ subjects received one dose of PsA-TT 10 µg at 9 to 12 months of age and EPI vaccines

^d^ subjects received one dose of PsA-TT 5 µg at 9 to 12 months of age and EPI vaccines

^e^ subjects received EPI vaccines, Measles and Yellow Fever vaccines at 9 to 12 months of age and Measles and Rubella vaccines at 15 to 18 months of age^.^

^f^Prior to first vaccination

^g^4 weeks after the first vaccination

^h^Second vaccination

^i^4 weeks after the second vaccination
